# Selective Release of Recombinant Periplasmic Protein From *E. coli* Using Continuous Pulsed Electric Field Treatment

**DOI:** 10.3389/fbioe.2020.586833

**Published:** 2021-02-09

**Authors:** Felix Schottroff, Jens Kastenhofer, Oliver Spadiut, Henry Jaeger, David J. Wurm

**Affiliations:** ^1^Institute of Food Technology, University of Natural Resources and Life Sciences (BOKU), Vienna, Austria; ^2^BOKU Core Facility Food & Bio Processing, Vienna, Austria; ^3^Research Division Biochemical Engineering, Integrated Bioprocess Development, Institute of Chemical, Environmental and Bioscience Engineering, TU Wien, Vienna, Austria

**Keywords:** continuous downstream processing, electroporation, host cell impurities, outer membrane, periplasmic protein, primary recovery, pulsed electric field, selective product release

## Abstract

To date, high-pressure homogenization is the standard method for cell disintegration before the extraction of cytosolic and periplasmic protein from *E. coli*. Its main drawback, however, is low selectivity and a resulting high load of host cell impurities. Pulsed electric field (PEF) treatment may be used for selective permeabilization of the outer membrane. PEF is a process which is able to generate pores within cell membranes, the so-called electroporation. It can be readily applied to the culture broth in continuous mode, no additional chemicals are needed, heat generation is relatively low, and it is already implemented at industrial scale in the food sector. Yet, studies about PEF-assisted extraction of recombinant protein from bacteria are scarce. In the present study, continuous electroporation was employed to selectively extract recombinant Protein A from the periplasm of *E. coli*. For this purpose, a specifically designed flow-through PEF treatment chamber was deployed, operated at 1.5 kg/h, using rectangular pulses of 3 μs at specific energy input levels between 10.3 and 241.9 kJ/kg. Energy input was controlled by variation of the electric field strength (28.4–44.8 kV/cm) and pulse repetition frequency (50–1,000 Hz). The effects of the process parameters on cell viability, product release, and host cell protein (HCP), DNA, as well as endotoxin (ET) loads were investigated. It was found that a maximum product release of 89% was achieved with increasing energy input levels. Cell death also gradually increased, with a maximum inactivation of -0.9 log at 241.9 kJ/kg. The conditions resulting in high release efficiencies while keeping impurities low were electric field strengths ≤ 30 kV/cm and frequencies ≥ 825 Hz. In comparison with high-pressure homogenization, PEF treatment resulted in 40% less HCP load, 96% less DNA load, and 43% less ET load. Therefore, PEF treatment can be an efficient alternative to the cell disintegration processes commonly used in downstream processing.

## Introduction

Periplasmic expression of recombinant proteins in *E. coli* has been investigated extensively in the past decades (Zhou et al., 2018; Sandomenico et al., 2020). The oxidative environment of the periplasm favors correct folding of proteins containing disulfide bridges and generally enhances solubility and stability of the product (Sandomenico et al., 2020). Translocation through the inner membrane is achieved by adding signal sequences initiating specific transport pathways to the product. Periplasmic expression has great potential for downstream processing because only the outer membrane (OM) has to be disrupted for extraction of the product and the periplasmic space contains less impurities, such as host cell protein (HCP) or DNA (Balasundaram et al., 2009a). However, selective disintegration of the OM is still hard to realize to date. Several approaches to make the OM “leaky” and selectively release the product from the periplasm have been reported in literature and are covered in extensive reviews (Balasundaram et al., 2009a; Kleiner-Grote et al., 2018). First, strains can be made leaky *in situ* on a genetic level for product release in the upstream process, for example, by knock-out of genes encoding structural cell envelope components and membrane proteins (Gao et al., 2018; Yang et al., 2018) or by inducible repression of cell proliferation (Kastenhofer et al., 2020). However, such strains may display reduced viability or require extensive optimization in the upstream process compared with industrial standard strains. Second, mechanical or non-mechanical methods can be applied to permeate the OM both in upstream (during cultivation) and in the downstream process (after harvest). Non-mechanical methods include (1) addition of chemicals like detergents, chaotropic agents, solvents, or acids (Wurm et al., 2017b; Schimek et al., 2020); (2) addition of lysozyme (Pierce et al., 1997); or (3) osmotic shock (Balasundaram et al., 2009a; Schimek et al., 2020). Although some of these methods show good selectivity, they are difficult to scale up due to the cost of chemicals or enzymes and the need to remove the additives in later downstream steps. Reported mechanical methods for periplasmic product release are ultrasonication or hydrodynamic cavitation (Balasundaram et al., 2009a; Eggenreich et al., 2017); however, their selectivity is still rather low and scale-up for sonication is hard to realize. Thus, high-pressure homogenization (HPH) remains the standard method for extraction of protein from *E. coli* cells (Eggenreich et al., 2020).

In this regard, pulsed electric field (PEF) may be a promising process to overcome some of the aforementioned limitations of protein recovery and purification. This treatment typically employs high voltage in the kilovolt range and associated electric field strengths of up to 40 kV/cm (Schottroff et al., 2017). Upon application of an external electric field, a transmembrane voltage is induced at the membrane of vegetative cells, which increases with increasing electric field strength. As a consequence, a shift of charges along the membrane takes place, accompanied by an accumulation of oppositely charged ions on both sides of the membrane. The occurring electro-compressive forces are proportional to the magnitude of the applied electric field strength, ultimately leading to a dielectric breakdown of the membrane and associated pore formation, the so-called electroporation (Sale and Hamilton, 1967; Zimmermann et al., 1974; Coster and Zimmermann, 1975; Neumann et al., 1982). The cell-specific threshold value for the occurrence of electroporation is called the critical electric field strength *E*_*crit*_ (Grahl and Märkl, 1996). For *E. coli*, *E*_*crit*_ values around 10 kV/cm are reported (Schottroff et al., 2017). Depending on the treatment intensity, electroporation can be either reversible or irreversible, i.e., the cell may or may not be able to repair the occurring damage, which is accompanied by the maintenance of physiological functions, or loss of viability and eventual lysis.

The increase of cell membrane permeability due to electroporation is used by a variety of applications in food and biotechnology, as well as in medicine. Exemplarily, PEF treatment is currently used for gene transformation and transfection in genetic engineering (Kumar et al., 2019; Ozyigit, 2020), pretreatment of plant tissues for mass transfer enhancement (Fauster et al., 2018; Ostermeier et al., 2018), microbial inactivation (Schottroff et al., 2019; Timmermans et al., 2019), or electrochemotherapy (Campana et al., 2019; Geboers et al., 2020). PEF applications reach from laboratory scale to industrial scale, with throughput levels of several tons per hour (Siemer et al., 2018). An extensive overview of applications based on electroporation is given by Kotnik et al. (2015) and Siemer et al. (2018).

Electroporation may also be deployed as a method to selectively extract valuable compounds from microorganisms, including yeasts, microalgae, and bacteria, although it is not industrially implemented yet (Martínez et al., 2020). Studies conducted on the extraction of protein from *E. coli* are summarized in Table 1. Although these contributions indicated the potential of PEF for selective protein release, the applicability for downstream processing remains unproven. Most studies employed batch-wise PEF treatment, which, in contrast to continuous PEF treatment, does not allow high throughput for large-scale industrial application due to limitations in generator power. Moreover, detailed analysis of impurity release (HCP, DNA, and endotoxins) is missing. Lastly, release efficiencies were low or cells were resuspended in additional buffers with various additives, which would require additional steps during downstream processing.

**TABLE 1:** Comparison of studies on PEF assisted protein extraction from *E. coli.*

*Modus operandi*	PEF matrix	Field strength (kV/cm)	Pulse frequency (Hz)	Pulse duration (μ s)	Energy input (kJ/kg)	Target protein	Investigated process variables	Comments	References
Batch	Buffers containing NaCl, glycine, and PEG	7.5–10	50	≤1	100–280	Recombinant β-glucosidase^*a*^, α-amylase^*b*^, and cellobiohydrolase^*a,b*^	Protein release	Up to 89% release of α-amylase in combination with NaCl and PEG	Ohshima et al., 2000
Batch	dH_2_O	5–20	1–1,000	100–1,000	5.5–533.8	Total protein^*a,b*^	Viability, protein release	∼75% of total protein extracted	Haberl-Meglič et al., 2015; Haberl-Meglič et al., 2016
Continuous (0.08 L/h)	dH_2_O, subsequent incubation in “post pulse buffers”	5.5–7.5	4	500–2,000	n.a.	Native PGK^*a*^ and GAPDH^*a*^	Viability, protein release, HCP impurity (qualitative)	Up to ∼90% release of native enzyme after incubation with specific buffers	Coustets et al., 2015
Continuous (0.6–1.98 L/h), recirculation to culture	Culture broth	12	2–3	n.a.	n.a.	Recombinant α-amylase^*b*^	Viability, protein release, HCP impurity (qualitative)	30% release of α-amylase using intermittent PEF treatment	Shiina et al., 2004, 2007
Continuous (1.5 L/h)	Culture broth	25.6–38.4	50–1,000	3	10.3–257.6	Recombinant Protein A^*b*^	Viability, protein release, HCP impurity (quantitative), DNA load, endotoxin load	Up to 89% release of recombinant periplasmic protein	This study

*^a^Located in the cytoplasm. ^b^Located in the periplasm.*

For the first time, this work shows the applicability of PEF-assisted selective extraction of recombinant periplasmic protein from *E. coli* for continuous biomanufacturing. The aim of this study was to maximize product release and minimize release of HCP, DNA, and endotoxins (ETs). Consequently, a setup operated in continuous mode for treatment of the culture broth was employed and the effect of specific energy input (*W*_*spec*_), pulse repetition frequency (*f*), and electric field strength (*E*) on cell viability, product release, and impurity load was evaluated. Finally, the parameter settings for the most efficient product release were determined and the results were compared with conventionally applied HPH. It was demonstrated that PEF treatment is a viable alternative to HPH for product recovery in continuous bioprocesses.

## Materials and Methods

### Strain, Media, and Cultivation

#### Strain

In this study, an *E. coli* BL21(DE3) strain overexpressing the IgG binding domain of *Staphylococcus aureus* Protein A (SpA) was used. The SpA gene was inserted on a pET30a plasmid and contained the N-terminal signal sequence pelB for translocation to the periplasm.

#### Media

For preculture and bioreactor cultivation, minimal medium according to DeLisa et al. (1999) was used. The carbon source was glucose, with concentrations 8, 20, and 400 g/L for the preculture, batch, and feed media, respectively.

#### Preculture

Preculture medium (500 ml) was inoculated with 1 ml of a frozen cell stock in a baffled shake flask. The preculture was incubated overnight at 37°C and 200 rpm in a shaking incubator (Infors AG, Bottmingen, Switzerland).

#### Bioreactor Cultivation

The bioreactor cultivation was conducted in a Techfors-S reactor (Infors AG, Bottmingen, Switzerland) with a working volume of 10 L. The reactor was continuously aerated at 10 L/min and stirred at 900 rpm and dissolved oxygen (DO) was kept above 40% by adding pure oxygen if needed. pH was controlled at 7.0 by addition of 12.5% (v/v) NH_4_(OH) and temperature was kept at 37°C unless stated otherwise.

Five hundred milliliters of the preculture was used to inoculate 4,500 ml of medium in the bioreactor. After the end of the batch phase that was detected by a spike in the DO signal, the feed was started and cells were grown to a cell dry weight (CDW) concentration of 35 g/L. Then, the temperature was lowered to 30°C and 0.5 mM isopropyl β-D-1-thiogalactopyranoside was added to induce heterologous gene expression. The feed rate was adjusted to an initial specific substrate uptake rate of 0.14 g_*glu*_/g_*CDW*_/h and kept constant for 8 h. After an 8 h induction phase, the cultivation broth was harvested via a draining valve and immediately cooled on ice for further processing and to prevent changes in membrane composition by reducing metabolic activity (Inouye and Phadtare, 2004). It can be assumed that this chilling step did not result in membrane disintegration (Cao-Hoang et al., 2010). The CDW concentration at the time of harvest (*c*_*Xharv*_) was gravimetrically determined to be 41.5 ± 0.16 g/L. The intracellular and extracellular SpA concentrations were 154 ± 10 mg/g_*CDW*_ and 82 ± 1 mg/g_*CDW*_, respectively, determined by HPLC (see Section “Analyses”).

### PEF Treatment

#### Setup and Continuous Trials

After 1–2 h of cooling on ice (∼2°C), the *E. coli* cultivation broth was continuously treated using PEFs. For this purpose, a semi–industrial-scale 6 kW generator (ScandiNova Systems AB, Uppsala, Sweden) was used, which is able to provide rectangular mono- or bipolar pulses, with a maximum magnitude of 25 kV, pulse widths of 0.5–5 μs, maximum current of 300 A, and a maximum pulse repetition frequency of 1 kHz. The use of pulses in the lower microsecond range enables the applicability of higher frequencies for a given level of energy input *W*_*spec*_.

**FIGURE 1:**
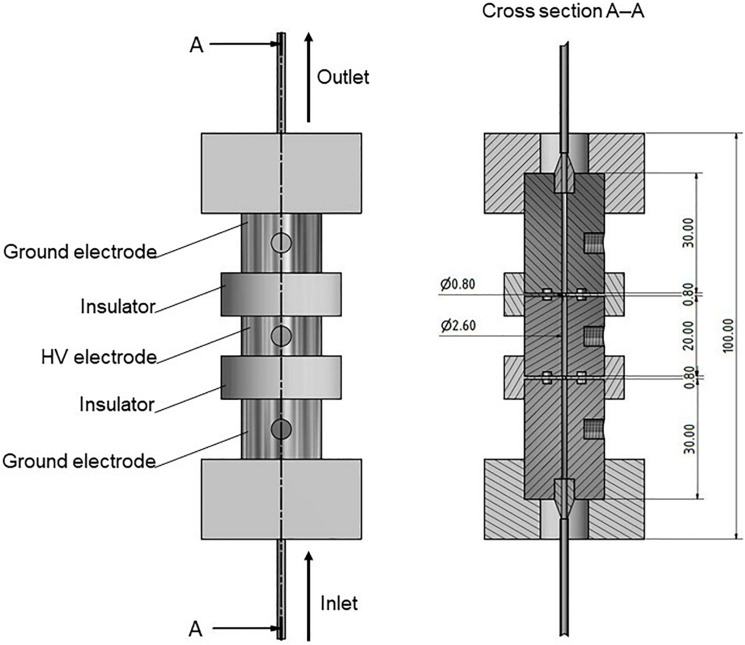
Drawing (left) and cross-section (right) of the used co-linear PEF treatment chamber. The chamber was held together by threaded rods through the top and bottom plates (not shown). High voltage is abbreviated by HV. All dimensions are given in mm.

The generator was connected to a specifically designed self-built co-linear treatment chamber, comprising stainless steel electrodes and polyoxymethylene insulators (see Figure 1). A peristaltic pump (MBR PP 101; MBR Bio Reactor AG, Wetzikon, Switzerland) was used to provide the desired mass flow of 1.5 kg/h and was connected to the treatment chamber by silicone tubes (d_*i*_ = 4 mm; d_*o*_ = 6 mm). The implementation of the peristaltic pump in combination with sterilized tubes also allows for a sterile operation, if necessary.

For continuous trials, the system was started using saline solution with the same electrical conductivity as the bacterial suspension (10 mS/cm). Once a steady state was reached, the saline solution was replaced by the culture broth containing the *E. coli* cells. After 10 min, an appropriate amount of treated suspension was sampled and electrical parameters were changed. After 5 min, the next sample was taken. Samples were immediately cooled on ice for 1–2 h before further processing, which can be assumed not to have a disruptive effect on the cell membranes (Cao-Hoang et al., 2010). This was repeated until all parameter combinations were accomplished. Sampling times were based on previously determined residence time distribution profiles (data not shown) and an additional margin of safety. The residence time of volume elements within the treatment zone (inside the insulators) was 1.9 ms. Untreated negative control samples were passed through the equipment in a comparable manner, but without exposure to the electric field.

The process was designed in such a way that a specific average field strength *E* (kV/cm), the pulse frequency *f* (Hz) as well as a desired specific energy input level *W*_*spec*_ (kJ/kg_*broth*_) were adjusted according to the experimental design (see section “Experimental Design”). To obtain *E*, the necessary voltage *U* (kV) to be adjusted at the generator was calculated according to Equation 1, taking into account a specific conversion factor *C*_*chamber*_ (1/cm). This conversion factor was determined based on a computational fluid dynamics simulation of the electric field distribution within the chamber (data not shown), as described elsewhere (Jaeger et al., 2009a). Briefly, ANSYS CFX 19.2 (ANSYS, Canonsburg, PA, United States) was used to solve a thermofluid dynamical model including equations for conservation of mass, momentum, energy, and electric charges. Boundary conditions were used as specified by Schottroff et al. (2020b), where further details considering the simulation and the underlying mathematical models can be found. For the present geometry, a *C*_*chamber*_ factor of 8.8/cm was determined. In comparison with the treatment chamber reported by Jaeger et al. (2009a), the chamber used in this study was down-scaled based on flow conditions by a factor of 5. The *C*_*chamber*_ factor given by Jaeger et al. (2009a) was 1.6/cm, corresponding to a relation of the two different *C*_*chamber*_ factors of 5.5-fold. Therefore, the obtained value is in accordance with the published literature.

(1) $$E=C_{chamber}*U$$

*W*_*spec*_ (kJ/kg) is calculated according to Eq. 2, from the applied voltage *U* (kV) (which can be expressed as *E*/*C*_*chamber*_ by rearranging Eq. 1), the current *I* (A), the pulse width *τ* (s), the pulse repetition frequency *f* (Hz), as well as the mass flow *ṁ* (kg/s). During each run, *τ* and *ṁ* were fixed, whereas *I* was measured. Thus, the desired energy input was obtained by adjustment of *U* (respective *E*) and *f*.

(2) $$W_{spec}=\frac{U*I*\tau *f}{\dot{m}}=\frac{E*I*\tau *f}{C_{chamber}*\dot{m}}=\Delta T*c_{p}$$

Temperature was measured directly before and after the treatment chamber, and the determined temperature increase Δ*T* (K) was correlated with the applied specific heat at constant pressure *c*_*p*_ [kJ/(kg K)] of the treated liquid (Eq. 2), to verify the validity of the applied processing conditions. For all reported trials, the initial sample temperature was set to 2°C, to reduce thermal load caused by the current flow during PEF treatment, especially for high-energy input levels. The resulting temperature increase levels are reported in Supplementary Table 1.

#### Experimental Design

**FIGURE 2:**
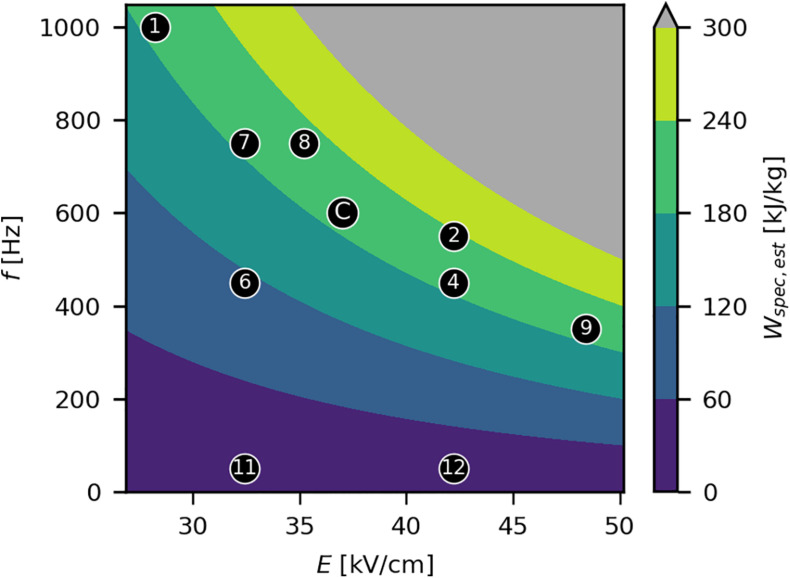
Experimental design for the PEF treatments and *W_*spec*__,est_* as a function of *E* and *f*. Black markers indicate the set points of *E* and *f*, with corresponding experiment numbers enclosed (Supplementary Table 1). The center point “C” was performed in triplicate. The gray area indicates technically infeasible parameter settings. *W_*spec*__,est_* was estimated assuming a constant *R* of 390 Ω. The reported levels of *W*_*spec*_ for the experiments were based on measurements of all quantities (Supplementary Table 1) and differed therefore slightly, due to temperature related fluctuations in *R*.

Two factors were considered for the modulation of *W*_*spec*_ in the experimental design: *E* and *f*. As the real *W*_*spec*_ could only be calculated from measured values of *I* and thus was unknown before the trials, a constant resistance (*R*) of the load of 390 Ω was used (based on preliminary trials; Supplementary Table 2) to calculate the estimated specific energy input *W_*spec*__,est_* during experimental design using Eq. 2 and Ohm’s law. The process conditions (*E* and *f*) were chosen in such a way to cover a wide range of realizable parameter combinations, also taking into account several parameter sets resulting in similar levels of *W*_*spec*_ (Figure 2). This allowed investigating the effect of *W*_*spec*_ and of the two factors *E* and *f*, independently of *W*_*spec*_, on the process performance. A fixed *τ* of 3 μs was chosen for all trials, based on a previous study (Schottroff et al., 2019), also given the narrow range of pulse widths provided by the generator. The mass flow *ṁ* was fixed at 1.5 kg/h to limit the degrees of freedom in the experimental design. Furthermore, the chosen *ṁ* lies within the typical range of laboratory-scale studies (Table 1) and was within the working range of the peristaltic pump used in this work. All factor combinations are listed in Supplementary Table 1. Due to temperature fluctuations, the real *R* (and thus *I*) during the experiments fluctuated between 350 and 474 Ω (Supplementary Table 1) and thus differed from the previously assumed *R*. The real values for *W*_*spec*_, calculated from measured *I*, were used for data evaluation.

### Analyses

PEF processed samples and the negative control were divided into 10 ml aliquots and centrifuged at 10,000 rcf for 10 min at 4°C. The biomass pellet and culture supernatant, hereafter referred to as PEF extract, were frozen in liquid N_2_ and stored at −20°C for later analysis. The reference method for product extraction was HPH (described later). For comparison with PEF treatment, the reference sample was the cell-debris-free homogenate of the culture broth from the negative control.

#### Viability

Subsequent to the treatments, PEF processed samples and the negative control were serially diluted in 1/4 strength Ringer’s solution (Merck KGaA, Darmstadt, Germany) and 50 μl of the appropriate dilutions was drop plated onto tryptic soy agar (TSA; VWR International SPRL/BVBA, Leuven, Belgium) plates in duplicate and incubated at 37°C for 24 h. Colony-forming units (CFUs) were manually counted and the corresponding inactivation levels were calculated from the initial bacterial concentration, *N*_0_ (CFU/ml), and the amount of viable cells after the treatment, *N* (CFU/ml), as log_1__0_(*N*/*N*_0_) (–). In the following paragraphs, log_10_ will simply be referred to as log. For all trials, the initial counts were in the range of 4.7 × 10^11^ CFU/ml, and the detection limit was 8.15 log.

#### Product Quantification

For extraction of the product remaining inside the cells, the biomass pellets were resuspended in 30 ml TE buffer (100 mM TRIS, 10 mM EDTA, pH 7.4), with the exception of the reference sample, which was processed as the original cell suspension in the culture broth. The suspension was homogenized in a high-pressure homogenizer (Emulsiflex C-3; Avestin, Ottawa, Canada) in three passages at 1,000 bar, which are optimal parameters for extraction of soluble product from *E. coli* according to Pekarsky et al. (2019). The homogenate was then centrifuged at 10,000 rcf (10 min, 4°C) to separate the cell debris from the soluble extract. SpA concentrations in the extracts from homogenization (intracellular, *c_*SpA*__,in_*) and from the PEF extract (extracellular, *c*_*SpA,ex*_) were then quantified in triplicate via reversed phase HPLC (Thermo Fisher Scientific, Waltham, MA, United States) using a polyphenyl column (Waters, Milford, MA, United States). The mobile phase consisted of a gradient of water and acetonitrile, supplemented with 0.1% trifluoroacetic acid. The released SpA (%) was then calculated according to Eq. 3.

(3) $$Proteinrelease=\frac{c_{SpA,ex}}{c_{SpA,in}+c_{SpA,ex}}*100$$

#### Protein Impurity Release

The release of host cell proteins by electroporation was measured in triplicate by SDS-PAGE. PEF extracts and reference sample were diluted 10× and 3×, respectively, in dH_2_O. Samples were further diluted in 2 × Laemmli buffer and incubated at 95°C for 10 min. Samples (10 μl) were then loaded onto precast SDS gels (4–15%, Mini-PROTEAN TGX; Bio-Rad, Hercules, CA), which were run at 180 V for 30 min. After staining with Coomassie Blue, the gels were imaged with a ChemiDoc system (Bio-Rad) and densitometric analysis was done with the GelAnalyzer software (version 19.1)^1^. The impurity load in percent was consequently calculated according to Eq. 4 using the area from the integrated curves of the densitograms:

(4) $$Impurityload=\frac{area_{impurities}}{area_{impurities}+area_{SpA}}*100$$

#### DNA Quantification

The PicoGreen assay kit (Thermo Fisher Scientific) was used for quantification of dsDNA in the PEF extracts, the negative control, and the homogenized reference sample. Triplicate measurements were done and SpA-specific DNA load (mg_*DNA*_/g_*SpA*_) was calculated according to Eq. 5:

(5) $$DNAload=\frac{c_{DNA}}{c_{SpA}}$$

where *c*_*DNA*_ (mg/L) is the concentration of dsDNA and *c*_*SpA*_ (g/L) is the concentration of SpA in the extract.

#### Endotoxin Quantification

The ET levels in the PEF extracts, the negative control, and the homogenized reference sample were measured with the ENDOLISA Kit (bioMérieux SA, Marcy-l’Étoile, France) according to the manufacturer’s instructions. Samples were diluted in ET free water by a factor 10^6^. The SpA-specific ET load (EU/g) was calculated with Eq. 6:

(6) $$ETload=\frac{c_{ET}}{c_{SpA}}$$

where *c*_*ET*_ (EU/L) is the concentration of ETs and *c*_*SpA*_ (g/L) is the concentration of SpA in the extract.

### Linear Regression

Multiple linear regression (MLR) was performed with the software MODDE 10 (Sartorius AG, Göttingen, Germany) to describe the investigated target variables [log(*N*/*N*_0_), product release, HCP load, DNA load, ET load] as functions of *E* and *f*. The model coefficients were determined with a significance level of α = 0.05 and corresponding values for “goodness of fit” *R*^2^ and “goodness of prediction” *Q*^2^ were calculated.

## Results and Discussion

This work aimed at the selective release of recombinant periplasmic protein from *E. coli* as a continuous product harvest step by PEF. A high extraction yield was targeted while keeping HCP, DNA, and ET loads to a minimum. For this purpose, the effect of electric field strength (*E*) and pulse repetition frequency (*f*), as well as the specific energy input (*W*_*spec*_), on the mentioned target variables as well as on cell viability was evaluated.

Initial parameter studies were carried out using electroporation cuvettes, to determine a suitable range of process parameters (*E* and *W*_*spec*_) for further investigation in a continuous process (data not shown). Likewise, it was investigated if the release efficiency of PEF treatment depended on biomass concentration (1–60 g_*CDW*_/L), which was not the case (data not shown). Therefore, undiluted *E. coli* suspensions (41.5 g_*CDW*_/L) from the bioreactor were used for PEF.

### Influence of Process Parameters on Cell Viability

**FIGURE 3:**
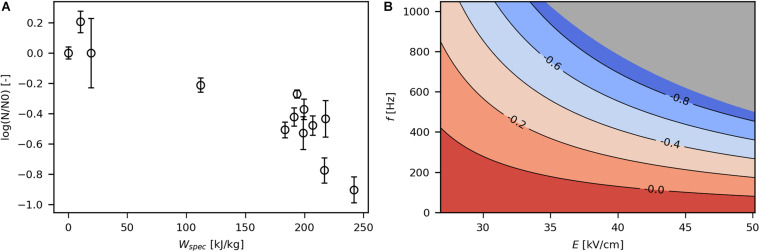
Effect of *W*_*spec*_ on log reduction **(A)** and contour plot showing the response of log reduction to *E* and *f* **(B)**. The underlying linear model of the contour plot had significant coefficients for *E*, *f* and E*f (α = 0.05), *R*^2^ was 0.82 and *Q*^2^ was 0.35. The gray area indicates technically infeasible parameter settings for the given PEF setup.

The impact of PEF treatment on viability was first assessed with respect to *W*_*spec*_, as it cumulates all relevant process parameters of a PEF treatment (Eq. 2). Viability of bacteria decreased with increasing *W*_*spec*_ (Figure 3A), as expected (Haberl-Meglič et al., 2015; Schottroff et al., 2019). In general, for all reported trials, log-reduction covered a range of no effect on viability (below 20 kJ/kg) to -0.9 log at the highest investigated energy input (241.9 kJ/kg), corresponding to 13% viable cells. Moderate *W*_*spec*_ (112.1 kJ/kg) resulted in −0.2 log (63% viability). Thus, the majority of the parameter space employed in this study led to a certain extent of irreversible electroporation.

The obtained linear regression models were used to construct contour plots that allow evaluation of the effect of *E* and *f* on the target variables. Figure 3B shows the contour plot for log reduction of viable cells. As expected, high *E* and *f* resulted in increased loss of viability because both parameters contribute to *W*_*spec*_. In fact, the influence of *E* and *f* on viability were interactive, so that the effect of one parameter on log reduction was enhanced with increasing levels of the other parameter and vice versa (see also Eq. 2).

Log reduction levels in the present study (down to -0.9 log) were low compared with usually targeted values for non-thermal inactivation of microorganisms by PEF (−5 to −7 log) (Food and Drug Administration, 2000). Comparably low overall inactivation levels might be due to the short electric field exposure times. Haberl-Meglič et al. (2015) employed similar *W*_*spec*_ and *f* as in the present study but applied pulse widths of 100–1,000 μs (compared with 3 μs in this study) and reported log-reductions of −1 to −3 log. Thus, the short pulse width applied in the present study might have prevented irreversible pore formation to an extent. Moreover, low inactivation may be explained by neutral pH during PEF treatment or high concentration of “solids,” which prevent inactivation of cells despite occurrence of electroporation (Jaeger et al., 2009b; Schottroff et al., 2020a).

During the exposure of the bacterial suspension to the electric field, there was a noticeable temperature increase (see Eq. 2 and Supplementary Table 1). Outlet temperatures ranged between 17.7 and 55.3°C, but samples were cooled within 13.6 s after the temperature increase caused by PEF. Thermal effects might affect viability, especially at high energy inputs and consequent high temperatures of up to 55°C. However, temperatures were elevated only for short time periods in this study. Using kinetics from the inactivation of *E. coli* in culture media (Stringer et al., 2000), it was estimated that in the worst case (constant temperature of 55.3°C for 13.6 s), the generated heat resulted in a viability reduction of -0.05 log. Therefore, it can be assumed that there was only a minor effect of temperature on cell inactivation during PEF treatment.

While high viability is generally not a prerequisite in downstream processing, product harvest by reversible electroporation is potentially interesting for process intensification. Continuous product extraction with subsequent recultivation of cells during upstream processing might allow to use the “cell factory” more efficiently (Shiina et al., 2004, 2007). The setup and parameter settings in the present study did not result in useful viability levels for this purpose (mostly below 63%). Further research is necessary to explore the potential of reversible electroporation by PEF for continuous product extraction.

### Influence of Process Parameters on Product Release

**FIGURE 4:**
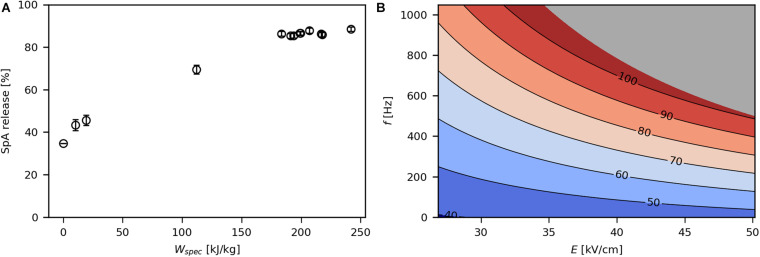
Effect of *W*_*spec*_ on SpA release **(A)** and contour plot showing the response of SpA release to *E* and *f* **(B)**. The underlying linear model of the contour plot had significant coefficients for *E*, *f*, and E*f (α = 0.05), *R*^2^ was 0.97 and *Q*^2^ was 0.93. The gray area indicates technically infeasible parameter settings for the given PEF setup.

In the control sample without PEF treatment, 35% of SpA were released into the culture medium (Figure 4A). This is due to the common phenomenon that the OM of *E. coli* becomes permeable during recombinant protein production, leading to protein leakage into the extracellular space (Han et al., 2003; Wurm et al., 2017a; Kastenhofer and Spadiut, 2020). The release of periplasmic product increased with increasing *W*_*spec*_ until a plateau was reached at around 89% released SpA. Above a *W*_*spec*_ of 180 kJ/kg, the release efficiency did not increase any further. This may be due to cytosolic SpA that could not permeate through both membranes upon PEF treatment. In this range, viability does not correlate with SpA release (Supplementary Figure 1), thus any increase in power input merely inactivates cells without enhancing product extraction.

The impact of *E* and *f* on SpA release is depicted in Figure 4B. Similar to the log reduction, the effect of each parameter on product release increased at higher levels of the other parameter. Haberl-Meglič et al. (2015) also showed that total protein release from *E. coli* is affected by *E*. However, in contrast to their study, the obtained data suggest that *f* has a much stronger impact on SpA extraction than *E* over the investigated design space. Because the used levels for *E* (28.2–48.4 kV/cm) are far above the critical electric field strength *E*_*crit*_ (∼10 kV/cm) for *E. coli* (Grahl and Märkl, 1996), *E* was likely high enough in all tested parameter combinations to evoke pore formation in all cells. Therefore, an increase in *E* might not have drastically improved electroporation. On the other hand, increasing the total field exposure time via *f* may result in sufficiently long periods of “open pores” allowing high amounts of protein to pass through the OM (Supplementary Figure 2). High levels of *f* are particularly necessary, when higher volumetric throughputs are desired, as otherwise a sufficient number of pulses per volume element is not given. This might be the reason for the low release efficiency of 30% in the studies of Shiina et al. (2004, 2007), who applied pulse repetition frequencies of 2–3 Hz (Table 1). Lastly, it could be shown that microsecond pulses can be efficiently used for the extraction of protein from *E. coli*. This is in contrast to the studies of Coustets et al. (2015) and Haberl-Meglič et al. (2015), who reported improved release efficiency at long pulse widths in the millisecond range, possibly due to lower values for *E* or *f* employed in their studies (Table 1).

### Influence of Process Parameters on Host Cell Impurities

**FIGURE 5:**
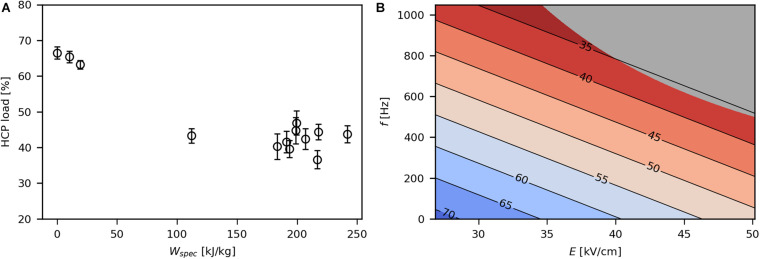
Effect of *W*_*spec*_ on HCP load **(A)**, expressed as percentage of HCP in the PEF extract, and contour plot showing the response of HCP load to *E* and *f* **(B)**. The underlying linear model of the contour plot had significant coefficients for *E* and *f* (α = 0.05), *R*^2^ was 0.79 and *Q*^2^ was 0.65. The gray area indicates technically infeasible parameter settings for the given PEF setup.

At low *W*_*spec*_ (below 20 kJ/kg), between 66 and 71% of extracellular proteins were HCPs, while higher *W*_*spec*_ (above 100 kJ/kg) resulted in a reduction of HCP load to levels between 32 and 47% (Figure 5A). Thus, high levels of *W*_*spec*_ led to a higher selectivity. This may be due to high intracellular product concentration (154 mg/g_*CDW*_) or small size (34 kDa) of the recombinant SpA compared with the HCP (Schimek et al., 2020). Both *E* and *f* had a significant effect on HCP load, which is due to their contribution to *W*_*spec*_ (Figure 5B). The origin of the HCP was not identified in the present study, although it may be assumed that periplasmic HCP was more prone to be released during PEF treatment than cytoplasmic protein (Ohshima et al., 2000). Yet it is likely that in the present study small amounts of cytoplasmic HCP were released as well, because DNA was also detected in the culture medium.

**FIGURE 6:**
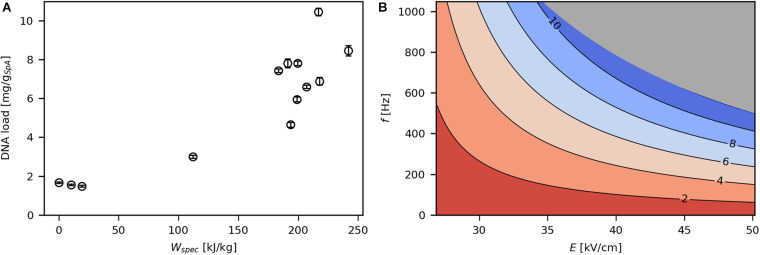
Effect of *W*_*spec*_ on SpA specific DNA load **(A)** and contour plot showing the response of product specific DNA load to *E* and *f* **(B)**. The underlying linear model of the contour plot had significant in coefficients for *E*, *f*, and E*f (α = 0.05), *R*^2^ was 0.95 and *Q*^2^ was 0.86. The gray area indicates technically infeasible parameter settings for the given PEF setup.

SpA-specific DNA load increased with rising levels of *W*_*spec*_ (Figure 6A) to a maximum of 10.5 mg/g_*SpA*_, which corresponds to 2.1 mg/g_*CDW*_ or 6.9% of the total DNA content of the *E. coli* cells (Neidhardt and Umbarger, 1996). The effects of *E* and *f* on DNA release (Figure 6B) were similar compared with viability and SpA release, such that the effect of one parameter on DNA leakage increased at higher levels of the other parameter and vice versa. As mentioned earlier, the release of DNA to the culture supernatant indicates pronounced pore formation in the IM. Interestingly, although cells seemed to be largely intact even at a high *W*_*spec*_ of 216.7 kJ/kg (6.9% of DNA released), only 17% of cells remained viable at that point. Thus, PEF treatment seems to be detrimental to physiological functions by disrupting membrane potential, pump activity as well as metabolic activity (Schottroff et al., 2017), while cells do not completely lyse.

**FIGURE 7:**
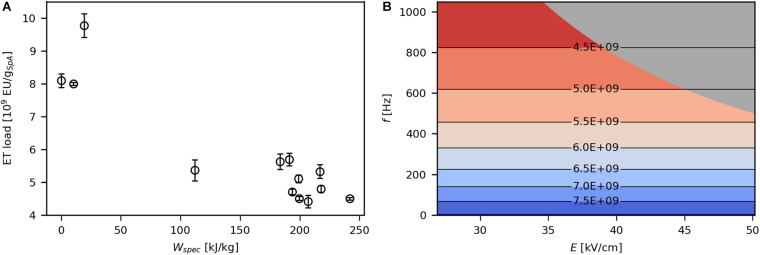
Effect of *W*_*spec*_ on product specific ET load **(A)** and contour plot showing the response of product specific ET load to *E* and *f* **(B)**. The underlying linear model of the contour plot had a significant coefficient for *f* (α = 0.05), *R*^2^ was 0.75 and *Q*^2^ was 0.56. The gray area indicates technically infeasible parameter settings for the given PEF setup.

ETs, mainly composed of lipopolysaccharides, are present in the outer leaflet of the OM and are continuously excreted by *E. coli* during cultivation. They elicit severe immune responses in humans, which is why they need to be almost completely removed from biopharmaceutical compounds (Carta and Jungbauer, 2010). Therefore, the present work aimed at minimal ET release during PEF processing. Product-specific ET load decreased with increasing levels of *W*_*spec*_ (Figure 7A), which is due to higher amounts of SpA released at high energy inputs. Correspondingly, ET impurity was significantly affected by *f* (Figure 7B) because this parameter has a strong impact on product release as well. Still, ET load was in the same order of magnitude (10^9^ EU/g_*SpA*_) at all investigated parameter settings and even in the untreated sample. Thus, in contrast to the proposal of Haberl-Meglič et al. (2015) that ET release during electroporation may be a problem linked to OM degradation upon cell death, obtained data show that ETs are present before PEF treatment, and various processing conditions have no significant impact on release of ETs, regardless of their effect on viability.

### Applicability of PEF for Continuous Downstream Processing

**FIGURE 8:**
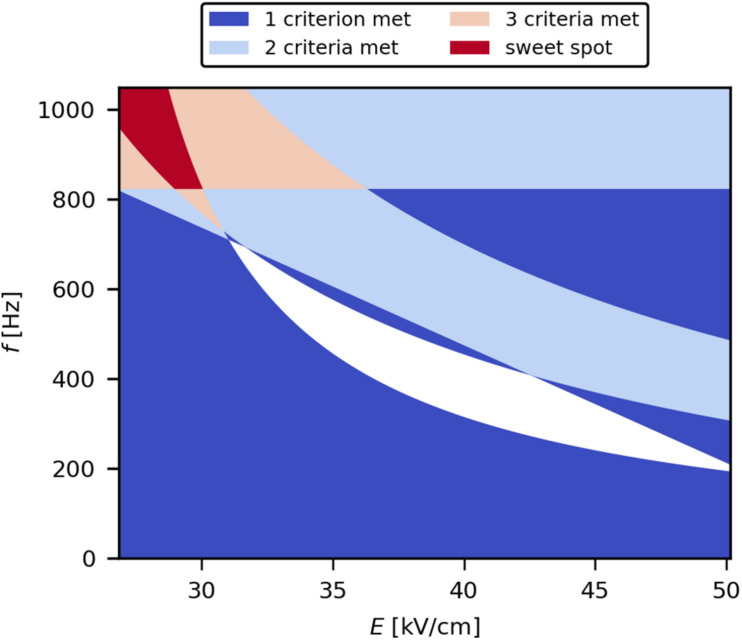
Sweet spot plot describing the parameter settings that result in the predefined criteria for SpA release (80–100%), HCP load (0–45%), DNA load (0–5 mg/g_*SpA*_), and ET load (0–4.5 × 10^9^ EU/g_*SpA*_).

Within the selected design space, the best settings of *E* and *f* were determined, resulting in maximal product release efficiency and minimal HCP, DNA, and ET loads, respectively (the so-called sweet spot). For this, the accepted ranges for the individual target variables were defined. They were chosen as an arbitrary range close to the theoretical maximum for product release and the theoretical minimum for HCP, DNA, and ET loads. The final ranges were 80–100% SpA release, 0–45% HCP load, 0–5 mg/g_*SpA*_ DNA load, and 0–4.5 × 10^9^ EU/g_*SpA*_ ET load. The obtained criteria were then imposed on the solutions of the model equations to retrieve the sweet spot plot (Figure 8). It was deduced that the sweet spot is achieved with *E* below 30 kV/cm and *f* above 825 Hz for the studied conditions (treated suspension with 10 mS/cm electrical conductivity, 3 μs pulse width, 1.5 kg/h mass flow). This can be explained by the fact that the used magnitudes of *E* were already sufficient for electroporation to occur; therefore, higher values of *E* likely did not increase the amount of electroporated cells, however may contribute to the formation of irreversible pores associated with cell death and lysis. On the other hand, as a flow-through process was used, higher levels of *f* allow the application of an increased number of pulses per volume element, thus increasing the electric field exposure time, resulting in high levels of protein release.

To show the benefit of PEF for periplasmic protein release, the results from an experimental point within the determined sweet spot (*E* = 28.2 kV/cm; *f* = 1,000 Hz) were compared with HPH (Table 2). Although HPH is an unselective method for the disintegration and subsequent extraction of proteins from cells, it is still commonly applied for the release of product from both cytoplasm and periplasm due to its high efficiency and scalability. While the release efficiency of PEF was 15% lower compared with HPH, there was a clear improvement in HCP and DNA loads. ET load was in the same order of magnitude for both processes (10^9^ EU/g). Considering the accepted ET levels in biopharmaceutical products of 10^1^–10^3^ EU/g (McCullough, 2011), similar steps would be required for ET reduction for samples treated with PEF or HPH. However, the DNA load was drastically reduced in the PEF process (by 96% relative to HPH) and would therefore mitigate the burden on the final product. In addition, the load of DNA, which has a strong negative charge, on the frequently used anion-exchange chromatography columns would be greatly reduced. Furthermore, low amounts of DNA also reduce viscosity-related issues in downstream processing (Balasundaram et al., 2009b; Carta and Jungbauer, 2010). Finally, the lower HCP impurity load after PEF-assisted product extraction (reduced by 40% relative to HPH) may reduce the number of purification steps needed.

**TABLE 2:** Comparison between HPH and PEF treatment for release of SpA.

	Log reduction	Product release	Impurity load		DNA load		ET load	
	(logN/N0)	(%)	(%)	Reduction (%)	(mg/g SpA)	Reduction (%)	(EU/g SpA)	Reduction (%)
HPH	−3^*a*^	100	69 ± 2	–	112.2 ± 8.7	–	8.2 ± 0.2 × 10^9^	–
PEF	−0.27	85 ± 1	41 ± 3	40 ± 4	4.7 ± 0.1	96 ± 0	4.7 ± 0.08 × 10^9^	43 ± 2

*^a^Assuming -1 log per passage at 1,000 bar (Wuytack et al., 2002).*

Regarding product stability and activity, both HPH and PEF may have detrimental effects on product quality to varying degrees. While HPH is characterized by cavitation and high shear forces, which may influence the protein structure (Vertessy et al., 2014; Han et al., 2020), the occurring temperature increase during the process may also be detrimental to the protein quality. The electric field present during PEF treatment, on the other hand, is reported to have limited effects on some proteins, especially enzymes with a metal ion in the prosthetic group (Castro et al., 2006). However, the accompanying temperature increase during the treatment seems to exert distinctly more pronounced effects (Jaeger et al., 2010). Therefore, outlet temperature should be carefully monitored and controlled during PEF treatment.

The continuous mode of operation of PEF further exemplifies the potential of its implementation in downstream processing, especially because such systems are scalable and already developed for other applications. The corresponding generator technology for large-scale production already exists, e.g., for food pasteurization with up to 5,000 L/h and a maximum power of 100 kW (Siemer et al., 2018). In comparison, HPH equipment with a similar throughput at 1,000 bar operates at twice the maximum power (200 kW) of a PEF generator (GEA, 2016). In terms of downstream applications related to PEF, a variety of future research needs may be addressed in further studies. This includes upscaling trials with higher throughput levels, considering transferability of results, and treatment homogeneity of larger systems. Moreover, only standard treatment chamber configurations were reported for PEF-assisted product recovery from microorganisms so far, although design and optimization of equipment may contribute to revealing the full potential of the technology for this application. Furthermore, other bacterial host organisms than *E. coli* as well as yeast cells, microalgae, and animal cell culture should be investigated to further evaluate the potential of continuous PEF treatment for product release.

## Concluding Remarks

In conclusion, it was shown that PEF is a useful process for the selective recovery of periplasmic proteins from *E. coli*. It was found that the investigated target variables (viability, product release, HCP, DNA, and ET loads) were mostly dependent on *W*_*spec*_, and the effects of the individual parameters *E* and *f* on these variables were similar to their contributions to *W*_*spec*_. Moreover, low electric field strengths (*E* < 30 kV/cm) and high pulse repetition frequencies (f > 825 Hz) were determined as the optimal parameter settings within the investigated design space, allowing efficient product release, while keeping the impurities to a minimum. With parameter settings within the sweet spot, a release efficiency of 85% and a significant reduction of HCP (40%), DNA (96%), and ET loads (43%) compared with HPH was achieved. Thus, PEF constitutes an interesting alternative for downstream applications in bioprocesses, as it can be readily applied to the culture broth in continuous mode.

## Data Availability Statement

The original contributions presented in the study are included in the article/Supplementary Material, further inquiries can be directed to the corresponding author/s.

## Author Contributions

FS and DW developed the idea for the study. FS, JK, and DW designed and performed the experiments and carried out analyses. FS and JK wrote the article. HJ and OS gave input toward the study. HJ, OS, and DW proofread the article. All authors contributed to the article and approved the submitted version.

## Conflict of Interest

The authors declare that the research was conducted in the absence of any commercial or financial relationships that could be construed as a potential conflict of interest.

## Footnotes

1 www.gelanalyzer.com

## Abbreviations

*E*: electric field strength (kV/cm)
ET: endotoxins
*f*: pulse repetition frequency (Hz)
HCP: host cell protein
HPH: high-pressure homogenization
IM: inner membrane
M: outer membrane
PEF: pulsed electric fields
SpA IgG: binding domain of *Staphylococcus aureus* Protein A
*W_*spec*_*: specific energy input (kJ/kg).
