# Supplementary material for: Selective Release of Recombinant Periplasmic Protein From E. coli Using Continuous Pulsed Electric Field Treatment
Source: Front Bioeng Biotechnol. 2021 Feb 9;8:586833. doi: 10.3389/fbioe.2020.586833 (PMC7900513; doi:10.3389/fbioe.2020.586833)
Supplement: Supplementary file 1 [file Data_Sheet_1.pdf]

Supplementary Table 1. Design of Experiments. Settings of voltage *U*, corresponding electric field strength *E*, pulse repetition frequency *f*, and corresponding measured current (I_{meas}), measured temperature increase (ΔT_{meas}; T_{inlet} = 2°C), and calculated specific energy input (W_{spec}) in the electroporation experiments. Fixed process parameters were mass flow $\dot{m}$ (1.5 kg/h) and pulse width τ (3 µs). The run order was randomized after experimental design. Experiments 3, 5, and 10 were replicates (marked as “C” in Figure 2 of the manuscript). In the negative control, the bacterial suspension was pumped through the device without applying voltage.

**run #**	**ctr**	**1**	**2**	**3**	**4**	**5**	**6**	**7**	**8**	**9**	**10**	**11**	**12**
U [kV]	0	3.2	4.8	4.2	4.8	4.2	3.7	3.7	4.0	5.5	4.2	3.7	4.8
E [kV/cm]	0	28.2	42.2	37.0	42.2	37.0	32.6	32.6	35.2	48.4	37.0	32.6	42.2
f [Hz]	0	1000	550	600	450	600	450	750	750	350	600	50	50
I_{meas} [A]	0	8.4	11.4	11	11.8	11.4	9.4	10	11.2	13.8	12	7.8	11.2
R_{meas} [Ω]	0	381	420	381	408	368	393	370	357	399	350	474	428
W_{spec} [kJ/kg]	0	193.5	216.7	199.6	183.5	206.8	112.1	198.7	241.9	191.3	217.7	10.3	19.4
ΔT_{meas} [K]	2	45.3	48.3	47.2	45.4	47.2	31.4	48.4	53.3	45.1	49.1	15.7	15.8

Supplementary Table 2. Preliminary continuous PEF trials for estimating the resistance *R*. The used set-up and fixed parameters were the same as during during the Design of Experiments. The material used for PEF treatment was *E. coli* broth (57 g/L cell dry weight) in minimal media, with electrical conductivity of 10 mS/cm, cooled to 2°C.

**run #**	**1**	**2**	**3**
U [kV]	3.7	4.2	4.8
E [kV/cm]	32.6	37.0	42.2
f [Hz]	450	600	550
I_{meas} [A]	9.3	11.2	12.1
**R_{meas} [Ω]**	**400**	**375**	**396**

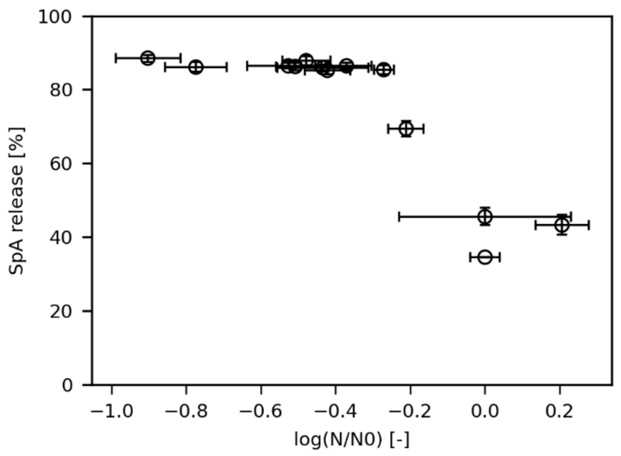

Supplementary Figure 1. Correlation between Protein A (SpA) release and log-reduction of viable *E. coli* cells.

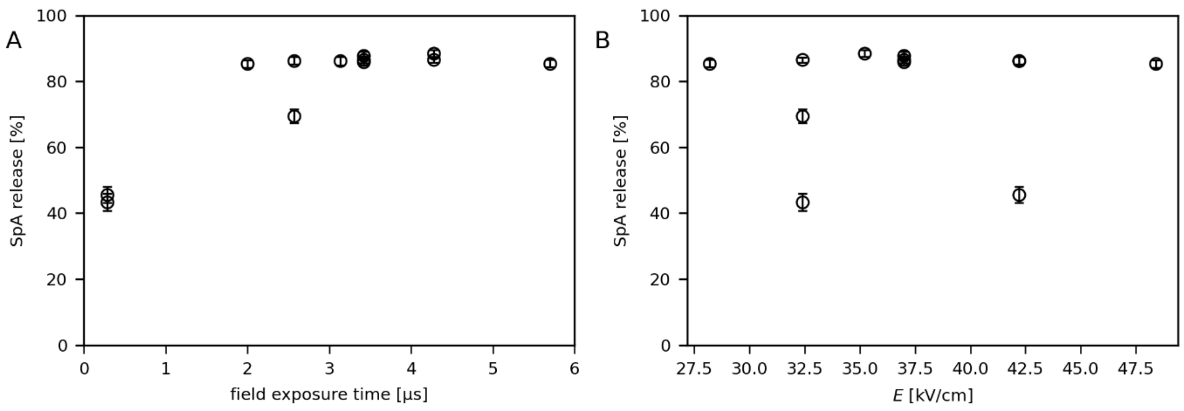

Supplementary Figure 2. The effect of total field exposure time (A) and electric field strength (B) on the release of Protein A (SpA).
